# Identification of Pain through Actigraphy-Recorded Patient Movement: A Comprehensive Review

**DOI:** 10.3390/bioengineering11090905

**Published:** 2024-09-10

**Authors:** Ricardo A. Torres-Guzman, Olivia A. Ho, Sahar Borna, Cesar A. Gomez-Cabello, Syed Ali Haider, Antonio Jorge Forte

**Affiliations:** 1 Division of Plastic Surgery, Mayo Clinic, 4500 San Pablo Rd., Jacksonville, FL 32224, USA; 2 Center for Digital Health, Mayo Clinic, Rochester, MN 55905, USA

**Keywords:** actigraphy, patient monitoring, physical activity, digital health, pain

## Abstract

Chronic pain affects over 50 million people in the United States, particularly older adults, making effective assessment and treatment essential in primary care. Actigraphy, which monitors and records limb movement to estimate wakefulness and sleep, has emerged as a valuable tool for assessing pain by providing insights into activity patterns. This review highlights the non-invasive, cost-effective nature of actigraphy in pain monitoring, along with its ability to offer continuous, detailed data on patient movement. However, actigraphy’s reliance on physical activity as a proxy for pain, and its inability to directly measure pain intensity, limit its applicability to certain pain types, such as neuropathic pain. Further research is needed to overcome these limitations and to improve the effectiveness of actigraphy in diverse clinical settings.

## 1. Introduction

The most frequent complaint in a primary care office is pain. In the United States, 20% of all patients, or more than 50 million people, experience chronic pain [1]. Older people are more likely to experience chronic pain than younger people [2]. It is crucial to treat a patient’s pain rationally and adequately due to the rise in opioid use disorder. The morbidity and mortality of our patients are directly impacted by how we evaluate pain. Providers should have various tools to define a patient’s pain to treat their symptoms better since over 30% of patients report pain lasting longer than six months. Chronic pain significantly reduces function and quality of life for 8% of adult patients and 6% of children [1,3]. Because pain is a subjective experience, many biopsychosocial components during the history-taking process are essential. Objective measures of pain, particularly chronic pain, aid in developing a standardized approach to orienting patients and providers to their pain to improve pain and patient outcomes such as function and quality of life. Many strategies have been proposed as possible solutions for measuring objective pain. Actigraphy is a method of recording and integrating the occurrence and intensity of limb movement activity over time. Actigraphy devices can be worn inconspicuously on the wrist, ankle, or waist for days to weeks. The devices are typically worn on the wrist or ankle for sleep applications. The data are then subjected to mathematical algorithms that estimate wakefulness and sleep. Actigraphy generates estimates of certain sleep parameters commonly estimated using sleep logs or measured directly by polysomnography (PSG), the gold-standard measure of sleep, and provides a graphical summary of wakefulness and sleep patterns over time [4]. This review aims to comprehensively review the literature to report and update on advances in objective pain assessment and monitoring, specifically, actigraphy to monitor and detect pain.

## 2. Clinical Use of Actigraphy

Actigraphy, first used to assess psychological disorders in the 1950s, has seen significant advancements [5]. Modern actigraphs are compact, dependable, and capable of recording and storing long-term patient activity levels. As a result, they are a valuable asset for sleep medicine clinicians because they can paint a picture of daily sleep–wake cycles and can be used to diagnose and evaluate sleep disorders and treatment outcomes [5].

The American Academy of Sleep Medicine concluded in 1995 that actigraphy could be used as a research tool, but its clinical utility was unknown. By 2002, the evidence had become weak enough to be used in clinical settings. The research literature supporting its use increased in 2007, particularly in evaluating circadian rhythm disorders, insomnia, hypersomnia, and obstructive sleep apnea. This resulted in the creation of a category 3 Current Procedural Terminology code in 2009 [5].

## 3. Pain Assessment

### 3.1. Overview of Pain Assessment Tools

Pain assessment is a critical step in the management of pain. It is important to accurately assess and monitor the intensity, duration, and type of pain experienced by a patient. There are various methods for assessing pain, including self-reports, behavioral observation, physiological measures, and psychophysical tests. Self-report assessments can include numerical rating scales, visual analog scales, verbal rating scales, and patient-specific questionnaires. Behavioral observation can include rating scales that measure facial expressions, body movements, or posture. Physiological measures include heart rate, respiration rate, skin conductance, and electromyography. Psychophysical tests include temporal summation tests, pressure algometry, and thermal stimulation. Each method has advantages and disadvantages, which must be considered when choosing which one to use.

Self-rating instruments are beneficial in helping physicians assess their patients’ health [6,7,8]. One-dimensional measures such as the Numeric Rating Scale (NRS) and visual analog scale (VAS) are available. The NRS is a straightforward tool in which patients rate their pain on a scale of 0 to 10, with 0 representing “no pain” and 10 representing “the worst pain imaginable”. It is easy to administer and widely used in both clinical and research settings due to its simplicity and quick assessment capability. The visual analog scale (VAS) operates similarly, using a 10 cm line where one end represents “no pain” and the other “worst pain imaginable”. Patients mark a point on the line that corresponds to their perceived pain intensity, which is then measured and quantified. Both the VAS and NRS are frequently used in clinical practice because they allow for quick and simple pain assessments.

When dealing with acute pain, these one-dimensional measurements can be adequate, but for chronic pain, the multidimensional nature of pain must be considered [9]. Multidimensional tools such as the McGill Pain Questionnaire (MPQ) and the Brief Pain Inventory (BPI) have been created. The MPQ is based on descriptive words which allow the patient to express their pain best [10]. The VAS and NRS are frequently used in clinical practice [6] but have certain limitations. These measures are subjective and may be affected by factors other than pain, such as mood [11,12,13]. This can lead to an inaccurate assessment of treatments’ effectiveness in clinical studies and daily practice. Furthermore, patients may base their pain scores on their own previously experienced pain, but this recall may not be precise [14]. Additionally, pain experiences differ between individuals, so the frame of reference differs for each person, resulting in different pain tolerances [15]. As the patient is the one indicating the pain score, it is impossible to blind them to the outcome of the VAS or NRS, giving them the ability to (unknowingly) contribute to the success or failure of therapy [16]. This brings up the issue of quantifying pain accurately. Therefore, there is a need for a valid, reliable, safe, and low-cost method to determine and quantify patients’ pain more objectively.

### 3.2. Overview of Pain Monitoring Wearables

Wearable technology is becoming more popular in the medical field because it allows accurate and efficient pain measurement. Wearables for pain’s objective measurement enable real-time tracking of pain levels, providing insights into treatment effectiveness and enabling more effective interventions. Furthermore, these devices can provide useful information about the intensity and duration of pain, allowing for a more precise and targeted treatment approach.

Wearable technology permits us to monitor physiological reactions connected to soreness, like heart rate, blood pressure, and breathing. This can aid us in comprehending the association between pain and the autonomic nervous system, which is triggered to protect from tissue damage and is linked to the central nervous system. Together with observations from the patient, this information may help us comprehend the duration and intensity of pain episodes in acute and chronic cases [17].

Nociception is a reliable method for detecting pain based on the autonomic nervous system’s response to a nociceptive stimulus. Because they are simple to obtain and sensitive to autonomic nervous system activity, heart rate variability and and plethysmography are commonly used in research and medical applications. The analgesia nociception index, based on heart rate variability, and the surgical pleth index, based on plethysmography, are the two most common medical devices used to measure pain [18].

There is a growing interest the shift of pain medicine from relying solely on subjective self-reported measures of pain towards using objective functional outcomes to inform treatment. While other medical fields have developed meaningful biomarkers and endpoints as objective measures, there have not been many successes in doing this with pain. fMRI has been able to detect painful stimuli with high sensitivity and specificity, though it is the least successful in detecting social pain. It is not practical to use imaging studies to measure pain in real time. Wearable accelerometers, video monitoring of facial expressions, and self-reported electronic surveys can be used as objective pain biomarkers. As a result, it is possible and necessary to supplement patient-reported pain assessments with objective measures of functional outcomes [17].

Currently, research in the field of pain and wearable devices is limited. Some studies have focused on comparing physical activity patterns measured by wearable accelerometers between pain patients and healthy individuals. A 2019 meta-analysis by Davergne et al. [19] found that wearable activity trackers significantly increased the number of steps and physical activity time, but long-term studies lasting over 8 weeks showed an increase in pain among patients. This meta-analysis included patients with various pain conditions and intervention strategies, offering new insights. Li et al. [20] discovered that using a wearable accelerometer combined with biweekly coaching improved physical activity in patients with knee osteoarthritis. In another study, Li et al. [21] examined the use of a wearable accelerometer with biweekly coaching by a physical therapist for 4 weeks and found no significant improvement in physical activity among patients with rheumatoid arthritis (RA) and systemic lupus erythematosus (SLE). However, a post hoc analysis revealed different outcomes for these subpopulations, with RA patients experiencing significant improvements in moderate-to-vigorous physical activity and pain reduction, while SLE patients did not. Similarly, Katz et al. [22] observed improvements in physical activity among RA patients using a combined behavioral and wearable accelerometer intervention. In contrast, McDermott et al. studied a combined behavioral and wearable accelerometer intervention in patients with peripheral arterial disease and found no significant effect on the number of steps taken or the distance covered in a 6 min walk test. McGovney et al. [23] discovered that in fibromyalgia patients with insomnia, increased morning and afternoon physical activity was associated with increased sleep disruptions, particularly in those with higher pain levels. These findings are consistent with the broader research, which indicates that while physical activity might be beneficial, its benefits can vary greatly depending on individual factors such as pain levels and the time of day.

The use of wearable devices for pain relief has been studied and has shown mixed results. Some studies found increased activity and pain reduction in specific pain subpopulations, while others found no significant improvement. When these findings are considered together, they point to a few possibilities. For starters, tailored behavior and wearable intervention strategies could be created for specific conditions. While the sample sizes in these studies were relatively small to moderate, which might result in misleading associations, exercise intervention feedback seems to be effective in enhancing physical activity and/or function in patients with rheumatoid arthritis and osteoarthritis, but not in those with systemic lupus erythematosus or peripheral arterial disease. Additionally, the behavioral components of these interventions vary between studies, complicating direct comparisons.

Recent studies have begun to explore the link between pain scores and wearable sensor data. A systematic review identified 16 studies since 2015, mostly utilizing accelerometer data, that investigated various populations and pain conditions (e.g., postoperative pain, sickle cell pain, arthritis pain, fibromyalgia, and critical illness). Murphy et al. [24] and Nørgaard et al. [25] found no association between average physical activity and pain in knee/hip osteoarthritis patients and juvenile idiopathic arthritis. However, Murphy et al. [24] observed a positive correlation between momentary activity and pain, suggesting that linking pain with accelerometry data could provide more insight than long-term averages or single-time-point analyses. Perraudin et al. [26] found that an unsupervised 5 Times Sit to Stand Test duration in the morning was significantly associated with pain intensity. Additionally, Patterson et al. [27] revealed that decreased postoperative activity was associated with a greater reduction in pain scores among patients who underwent knee and hip replacements.

## 4. Validation of Actigraphy Monitoring for Pain Monitoring Detection

### 4.1. Pain Monitoring

**Figure 1:**
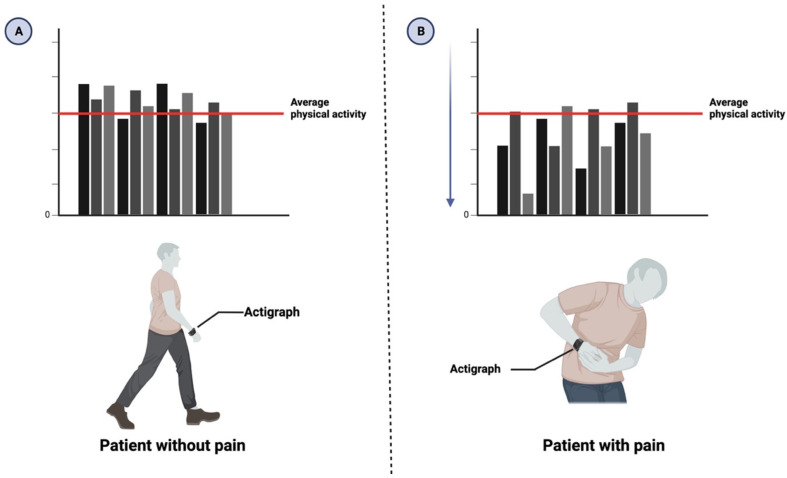
(**A**) Actigraphy is a method of recording physical activity. Patients not in pain are expected to have average physical activity compared to their baseline. (**B**) On the other hand, patients with pain are expected to have a different pattern of physical activity than their baseline in the graph bar, showing a decrease in their physical activity.

Actigraphy monitors are equipped with an omnidirectional accelerometer that records any physical activity, including the quantity and strength. The device contains a sensor that creates a voltage each time there is a change in acceleration in any direction and its strength (see Figure 1).

The hip-mounted monitor is widely recognized as the most precise tool for determining physical activity and energy expenditure compared to wrist- or ankle-mounted monitors [28].

### 4.2. Physical Activity and Prediction of Pain

A recent study by Kashikar-zuck et al. [29] found that physical activity levels of adolescents with juvenile primary fibromyalgia syndrome (JPFS) were correlated with parent-reported physical functioning and depressive symptoms, indicating that further study of the factors that predict perceived and actual physical functioning in JPFS is needed. Sabiston et al. [30] found that pain among survivors of breast cancer was associated with depression symptoms and negative affect and was inversely associated with positive affect. Additionally, physical activity was found to be partially responsible for mediating the relationship between pain and depression and pain and positive affect, suggesting that physical activity can help survivors of breast cancer to better manage pain and improve their mental health.

Naugle et al. [31] and Rabbitts et al. [32] conducted studies to explore the relationship between physical activity and pain modulatory function in older adults and adolescents respectively. Rabbits et al.’s study involving 119 adolescents aged 12 to 18 showed that higher pain intensity was associated with lower peak physical activity levels the next day, and higher mean physical activity levels predicted lower pain intensity ratings at the end of the day for those with chronic pain, suggesting that physical activity may be an important focus in chronic pain treatment. Naugle et al.’s study [31] involving 51 older adults revealed that sedentary time and light physical activity were associated with greater pain inhibitory capacity, while moderate-to-vigorous physical activity was associated with less pain facilitation, indicating that different types of physical activity behavior may have different impacts on pain modulation in older adults.

Kichline et al. [33] conducted a study investigating the physical activity levels of youths with chronic abdominal pain and the relationship between day-level physical activity and next-day pain intensity. They found that the youths did not meet the recommended levels of physical activity, and there was a negative effect of within-person total sleep time as a predictor of pain severity. Zhaoyang et al. [34] and Gavilan-Carrera et al. [35] explored the associations between sedentary behavior (ST) and physical and psychological health outcomes. Zhaoyang et al. [34] found that reducing sedentary behavior could help improve the physical and emotional well-being of those with chronic abdominal pain and osteoarthritis. Gavilan-Carrera et al. [35] found that reducing ST and increasing light physical activity (PA) and moderate-to-vigorous physical activity (MVPA) was associated with better bodily pain and the Short Form-36 (SF-36) physical component at 5-year follow-up, and increasing MVPA was associated with less pain and a better SF-36 physical component at 2- and 5-year follow-up. These findings suggest that reducing daily sedentary behavior and increasing physical activity could help improve physical and emotional well-being in those with chronic pain and other conditions. Further research is needed to better understand the random effects pertaining to the relationship between physical activity and pain severity.

### 4.3. Pain Intensity and Prediction of Pain

A recent study conducted by Abeler, K. et al. [36] investigated the relationship between chronic primary musculoskeletal pain (CPMP), sleep quality, and mental distress in 56 patients over two seven-day data-collection periods in summer and winter. The results revealed that pain significantly affected sleep quality and marginal sleep duration, while sleep quality tentatively predicted next-day pain. Mental distress was the strongest predictor of pain but did not modify the sleep–pain associations or seasons. Similarly, Merkus, S.L. et al. [37] examined the association between neck and shoulder pain intensity (NSPi) and the composite metric of arm elevation and trapezius activity (neck/shoulder load) among 118 construction and healthcare workers over 2 years. It was found that time spent in 0.5–7.0% maximum voluntary effort (MVE) had the largest association with changes in NSPi. Furthermore, among those pain-free at baseline, medium and high neck/shoulder load contributed the most to explaining changes in NSPi. However, the neck/shoulder load composite metric did not show a stronger association with the course of NSPi than arm elevation or trapezius activity alone. These findings suggest that CPMP, sleep quality, and mental distress are closely related and should be considered when assessing and treating chronic pain patients.

### 4.4. Miscellaneous Factors That Affect Pain Prediction

Sarwar et al. [38] investigated machine learning and rest–activity circadian rhythm features in patients to predict chronic pain passively. The proposed PainRhythms approach achieved impressive results, with an average AUC-ROC of 0.97 for predicting pain, 0.67 and 0.62 for pain intensity and interference, and 0.56 for physical function, using actigraphy data to extract features such as activity, sleep, and Intradaily Variability (IV). Notably, IV was the most predictive feature, with higher pain values associated with disturbed sleep. While this study provides promising evidence of the ability of rest–activity rhythms to detect chronic pain, more research is needed to confirm these findings on a larger, more diverse, and class-balanced dataset.

Kiernan et al.’s [39] study also demonstrated the utility of activity monitors in predicting injury. Their research found that injured runners have significantly higher peak vertical ground reaction forces (vGRFs) and weighted cumulative loading (sum of peak vGRFs weighted to the ninth power) than uninjured runners. The data were collected using a hip-mounted activity monitor to quantify the loading profiles (load magnitude–number combinations) for nine collegiate athletes over a 60-day period across 419 runs. According to the findings of this study, activity monitors can be used to test hypotheses, develop injury prediction models, and design and adjust athlete- and loading-specific training programs and feedback (see Table 1).

## 5. Efficacy and Drawbacks of Actigraphy for Pain Monitoring

Actigraphy offers a promising, non-invasive method for monitoring pain by continuously tracking patient movement and activity levels [23,29,30,31,32,33,34,35,36,37,38,39]. Unlike traditional pain assessment tools such as the Numeric Rating Scale (NRS) and visual analog scale (VAS) which directly measure pain intensity, actigraphy provides insights into how pain may influence general activity and sleep–wake cycles [40]. This method focuses on capturing objective data about physical movement and sleep patterns, offering a distinct perspective on the impact of pain on daily functioning. 

However, actigraphy has significant limitations. Its primary drawback is the inability to directly measure pain intensity; instead, it relies on changes in physical activity as a proxy for pain. This approach may not accurately capture all pain types, particularly neuropathic pain, which might not manifest through noticeable changes in activity levels. For example, studies by McGovney et al. [23] and Kashikar-Zuck et al. [29] reveal that actigraphy may fail to provide a comprehensive assessment of how pain affects an individual’s daily functioning and sleep quality. Additionally, factors such as device placement and individual differences in physical activity patterns can introduce variability and affect the accuracy of the data collected.

Given these challenges, there is a clear need for further research to explore the full potential and limitations of actigraphy in pain assessment. Future studies should focus on improving the sensitivity of actigraphy in detecting pain-related activity changes, especially for conditions where movement is less affected. Integrating actigraphy data with other physiological and subjective pain measures, as suggested by studies such as those by Naugle et al. [31] and Rabbitts et al. [32], could enhance the accuracy and reliability of pain assessment tools. This multimodal approach is crucial for capturing the full spectrum of pain’s impact on individuals, addressing actigraphy’s limitations in measuring pain directly.

Expanding the use of actigraphy across diverse clinical settings and patient populations will be essential for validating its effectiveness. Moreover, advancements in machine learning and data analysis hold promise for refining the interpretation of actigraphy data, potentially leading to more personalized and precise pain management strategies [41,42]. For instance, the integration of machine learning models, as explored by Sarwar et al. [38], could improve the interpretation of complex data patterns related to pain, thereby enhancing actigraphy’s utility in clinical practice.

## 6. Summary and Conclusions

Pain is a frequent complaint in primary care. The improper evaluation and treatment of pain can lead to opioid use disorder and increased morbidity and mortality in patients. Actigraphy is a method of recording and integrating the occurrence and intensity of limb movement activity over time. It can estimate wakefulness and sleep and generate graphical summaries of these patterns. We can conclude that actigraphy is a promising tool for objectively assessing and monitoring pain. Its advantages over traditional pain assessment methods make it an attractive choice for clinicians and researchers. Its non-invasive nature and graphical representation of wakefulness and sleep patterns make it attractive for pain monitoring and assessment. Further studies are needed to elucidate the clinical utility of actigraphy in pain assessment and monitoring. Additionally, further research is needed to evaluate actigraphy’s potential limitations or drawbacks and its potential applications in other clinical settings.

## Figures and Tables

**Table 1:** Study characteristics and outcomes of using actigraphy to monitor/predict pain.

Ref.	Total Sample Size	Sex	Age (Years)	Study Population	Type of Pain	Device	Location of Actigraph	Comparison Methods	Main Findings
[29]	104	84% female	11 and 18	Juvenile primary fibromyalgia syndrome	Musculoskeletal	Not reported	Hip	VAS and FDI	Adolescents’ self-reports of pain, depressive symptoms, and functional disability showed no significant correlation with physical activity levels. Vigorous physical activity did not influence pain perception significantly.
[30]	145	100% female	28 to 79 (mean = 54.86, SD 10.83)	Survivors of breast cancer	Stomach pain, back pain, joint pain, pain during sexual intercourse, headaches, and chest pain	GT3M accelerometer (Actigraph, Pensacola, FL, USA)	Waist	Primary Care Evaluation of Mental Disorders screening questionnaire	Pain predicted higher depression symptoms and lower positive affect. Physical activity partially mediated the relationship between pain and both depression and positive affect.
[32]	119	71% female	12 to 18	(59 with chronic pain and 60 healthy)	Chronic pain	Actiwatch (Actiwatch 64; Phillips Respironics, Bend, OR, USA)	Wrist	11-point numerical rating scale (NRS)	Higher pain intensity linked to lower physical activity levels the following day. Higher medication use predicted lower physical activity on the same day. Increased physical activity was associated with reduced pain at the end of the day in adolescents with chronic pain.
[31]	51	Not reported	60 to 77	Healthy individuals	Not reported	Actigraph GT3X1	Hip	Pain Catastrophizing Scale	Different types of physical activity may influence pain inhibition and facilitation processes in older adults.
[39]	9	Not reported	Not reported	Injured runners	Musculoskeletal	Not reported	Hip	Pain/fatigue scale	No significant difference in pain/fatigue between injured and non-injured participants. High subjective pain/fatigue did not predict running-related injuries (RRIs).
[33]	71	74.6% female	8 and 17 (mean = 13.34, SD 2.67)	Chronic abdominal pain	Chronic abdominal pain	ActiGraph wGT3X-BT accel- erometer (Actigraph LLC, Pensacola, FL, USA)	wrist	5-point scale with the stem, “Do you have abdominal pain right now?” Participants responded using the following anchors: (1) no, none; (2) yes, minimal; (3) yes, mild; (4) yes, moderate; or (5) yes, severe.	Previous day’s moderate-to-vigorous physical activity (MVPA) affected pain severity. Total sleep time had a small negative impact on pain severity.
[34]	143	58% female	Mean = 65.39, SD 9.53	Knee OA	Knee pain	GT1M or GT3X model of the CSA/MTI triaxial ActiGraph	Hip	RADAR questionnaire + separate ratings were made for multiple joints or joint groups (i.e., knees, shoulders, hips, ankles, ball of foot, toe knuckles, elbows, wrists, hand knuckles, finger knuckles) on a scale from 0 to 3 (0 = no pain/ten- derness; 3 = severe pain/tenderness). Items were averaged to create a mean score for patients’ pain	Sedentary behavior predicted less pain but worse affect at the end of the day. Increased sedentary time was associated with worse next-morning affect.
[36]	56	72% female	18 to 65	Chronic primary musculoskeletal pain	Chronic primary musculoskeletal pain	Actiwatch Spectrum Plus device	Wrist	BPI	Pain was a significant predictor of next-night sleep quality. Sleep quality was marginally predictive of next-day pain.
[37]	121	60% female	Mean = 42.0, SD 12.0	Neck and shoulder pain	Neck and shoulder pain	Actigraph GT3X+, Actigraph, Florida, USA	Shoulder	NSPi during the past four weeks was reported by the workers on a 4-point scale from 0 ‘no pain’ to 3 ‘severe pain’. One question considered pain in the neck and another considered pain in the dominant shoulder. The anatomical areas were illustrated by a mannequin. NSPi, defined as the higher of the two scores, was recorded at base- line and every 6 months during the 2-year follow-up.	Neck/shoulder load composite metric did not strongly correlate with pain progression compared to individual metrics like arm elevation or trapezius activity.
[35]	427	Only females	51.4 ± 7.6 years	Fibromialgia	Global pain	GT3X+ Actigraph	Hip	FIQR-pain	Decreased sedentary time and increased light physical activity associated with better physical component and pain scores. Increased moderate-to-vigorous physical activity linked to reduced pain and better physical health after follow-up.
[38]	52	Not reported	Not reported	Chronic pain	Chronic pain	ActiGraphy GT3X+ device	Wrist	PROMIS-29 v2.0	Rest–activity rhythms (RARs) effectively predicted chronic pain and pain intensity. Machine learning models using RAR features showed high accuracy in predicting pain.
[23]	134	94% female (126 females, 8 males)	Mean age = 52 years, SD = 12 years, range = 18–77 years	Fibromyalgia and insomnia complaints	Chronic pain	Actiwatch-2 and AURA Portable Recording System for polysomnography	Wrist	0 to 100 Numerical Rating Scale	Greater morning and afternoon physical activity were associated with greater sleep disturbances (e.g., higher WASO and % stage 1 sleep). The association between afternoon physical activity and greater % stage 1 sleep was more pronounced in individuals with higher pain. No significant associations were found between late evening activity and sleep outcomes.

Abbreviations: BPI: Brief Pain Inventory; FDI: Functional Disability Inventory; FIQR-pain: visual analog scale and Fibromyalgia Impact Questionnaire; MVPA: moderate-to-vigorous physical activity; NSPi: pain intensity in the neck and in the dominant shoulder; PROMIS: Patient-Reported Outcomes Measurement Information System; PA: light physical activity; RADAR: The Rapid Assessment of Disease Activity in Rheumatology; RARs: rest–activity rhythms; SB: sedentary behavior; SF-36: 36-item short-form health survey; ST; sedentary time; VAS: visual analog scale.

## Data Availability

Not applicable.
